# Supplementary material for: Mutations in PCYT2 disrupt etherlipid biosynthesis and cause a complex hereditary spastic paraplegia
Source: Brain. 2019 Oct 22;142(11):3382–97. doi: 10.1093/brain/awz291 (PMC6821184; doi:10.1093/brain/awz291)
Supplement: awz291_Supplementary_Data [file awz291_supplementary_data.zip › awz291-Suppl_data/Supplementary_Data4.zip!/Supplementary information 4 - plasma lipidomics.zip!/Plasma lipidomics/Control vs Patient3a/Statistical plots and data/Volcano plot_alternative.pdf]

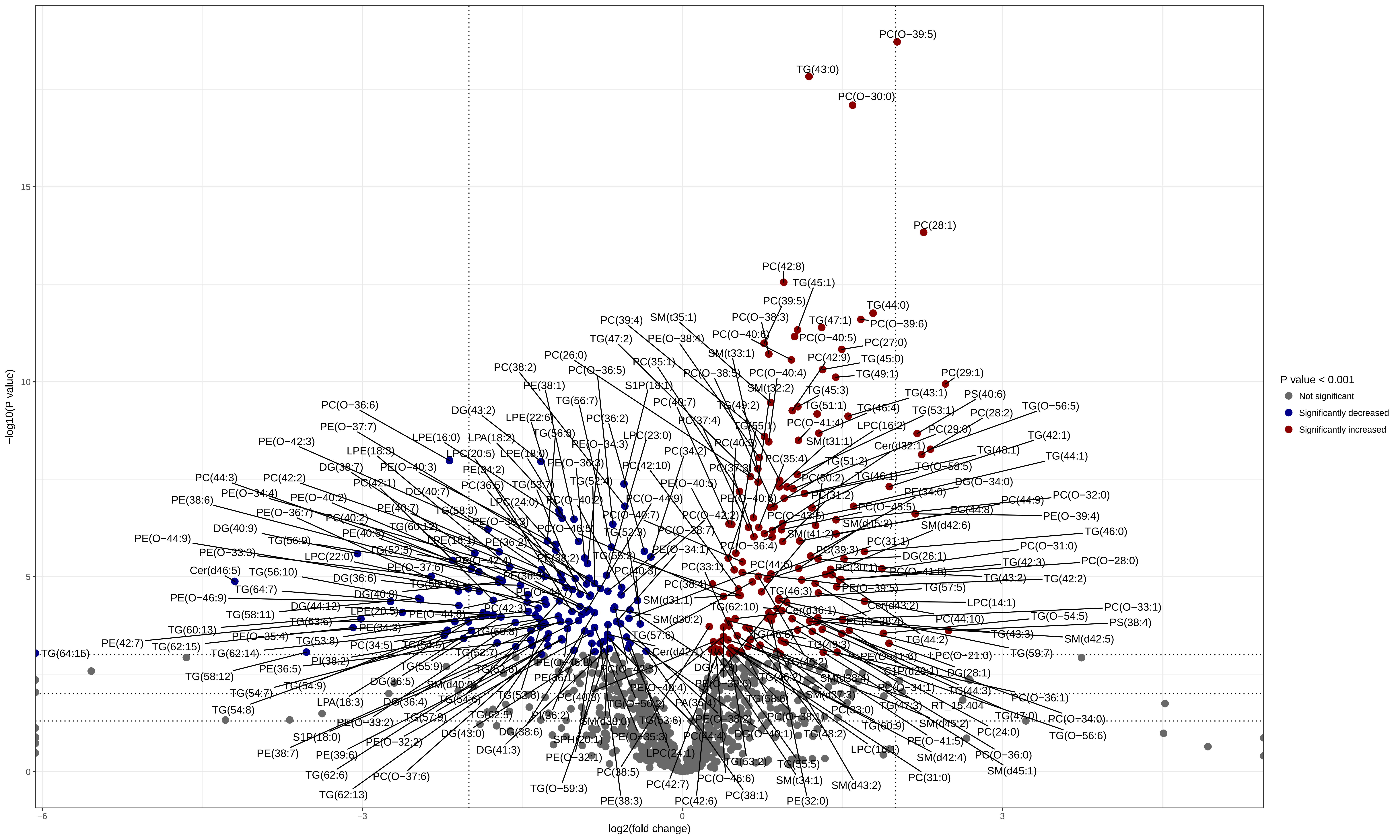

−log10(P value)
log2(fold change)
15
10
5
0
−6
−3
0
3
P value < 0.001
Not significant
Significantly decreased
Significantly increased
PC(O−39:5)
TG(43:0)
PC(O−30:0)
PC(28:1)
PC(42:8)
TG(45:1)
PC(39:5)
TG(44:0)
PC(O−38:3)
TG(47:1)
PC(O−39:6)
PC(39:4)
SM(t35:1)
PC(O−40:6)
PC(O−40:5)
TG(47:2)
PE(O−38:4)
PC(27:0)
SM(t33:1)
PC(42:9)
TG(45:0)
PC(26:0)
PC(35:1)
PC(38:2)
PC(O−36:5)
PC(O−38:5)
PC(O−40:4)
TG(49:1)
PC(29:1)
S1P(18:1)
SM(t32:2)
PE(38:1)
TG(45:3)
TG(43:1)
PS(40:6)
TG(56:7)
PC(40:7)
TG(49:2)
TG(51:1)
TG(46:4)
TG(53:1)
PC(28:2)
TG(O−56:5)
PC(O−36:6)
DG(43:2)
LPE(22:6)
PC(36:2)
PC(37:4)
PC(O−41:4)
TG(55:1)
LPC(16:2)
PC(29:0)
PE(O−37:7)
TG(56:8)
LPC(23:0)
SM(t31:1)
TG(42:1)
LPE(16:0)
LPA(18:2)
PE(O−34:3)
PC(40:5)
Cer(d32:1)
PE(O−42:3)
LPE(18:3)
LPC(20:5)
LPE(18:0)
PC(34:2)
PC(35:4)
TG(48:1)
TG(51:2)
TG(44:1)
DG(38:7)
PE(O−40:3)
PE(34:2)
PE(O−36:3)
PC(42:10)
PC(37:3)
TG(O−58:5)
PC(44:3)
PC(42:2)
PC(42:1)
PC(36:5)
TG(53:7)
TG(52:4)
PE(O−40:5)
PC(30:2)
TG(46:1)
DG(O−34:0)
PE(O−34:4)
DG(40:7)
PE(34:0)
PE(38:6)
PE(O−40:2)
LPC(24:0)
PC(O−40:2)
PC(O−44:9)
PE(O−40:6)
PC(31:2)
PC(44:9)
PC(O−32:0)
PE(40:7)
TG(58:9)
PC(O−45:5)
PC(44:8)
PE(O−36:7)
PC(40:2)
PE(O−38:3)
PC(O−40:7)
PC(O−42:2)
PC(O−43:5)
PE(O−39:4)
TG(60:12)
SM(d45:3)
DG(40:9)
PE(40:6)
PC(O−46:5)
TG(52:3)
PC(O−38:7)
SM(t41:2)
SM(d42:6)
PE(O−44:9)
TG(56:9)
LPE(18:1)
PE(36:2)
TG(46:0)
PE(O−33:3)
TG(52:5)
PE(O−34:1)
PC(O−36:4)
PC(31:1)
LPC(22:0)
DG(26:1)
PC(O−31:0)
PE(O−42:4)
TG(53:2)
PC(39:3)
Cer(d46:5)
TG(56:10)
PE(O−37:6)
PE(38:2)
PC(33:1)
PC(44:6)
PC(30:1)
TG(42:3)
PC(O−28:0)
DG(36:6)
TG(58:10)
PE(36:3)
PC(40:3)
PC(O−41:5)
TG(43:2)
TG(42:2)
TG(64:7)
PC(38:4)
PE(O−39:5)
TG(57:5)
DG(40:8)
PE(O−44:2)
TG(46:3)
PE(O−46:9)
SM(d31:1)
LPC(14:1)
DG(44:12)
TG(62:10)
Cer(d43:2)
PC(O−33:1)
TG(58:11)
LPE(20:5)
PC(42:3)
Cer(d36:1)
TG(O−54:5)
TG(63:6)
PE(O−44:8)
SM(d30:2)
PC(44:10)
PS(38:4)
TG(60:13)
PE(34:3)
TG(55:8)
PC(O−38:4)
PE(O−35:4)
TG(57:6)
TG(46:6)
TG(43:3)
TG(53:8)
SM(d42:5)
PE(42:7)
TG(62:15)
PC(34:5)
TG(54:5)
TG(49:3)
TG(44:2)
TG(64:15)
TG(62:14)
TG(52:7)
Cer(d42:1)
PE(O−41:6)
LPC(O−21:0)
TG(59:7)
PI(38:2)
PE(O−46:8)
TG(45:2)
TG(55:9)
DG(42:0)
S1P(20:1)
DG(28:1)
PE(36:5)
TG(52:6)
PC(O−42:3)
TG(58:12)
PE(36:1)
TG(46:2)
SM(d38:4)
DG(36:5)
SM(d40:0)
PE(O−39:6)
PC(O−34:1)
TG(54:7)
TG(54:9)
PE(O−46:4)
SM(d37:3)
TG(44:3)
PC(O−36:1)
TG(52:8)
PC(40:8)
TG(58:6)
TG(47:3)
RT_15.404
LPA(18:3)
DG(36:4)
TG(54:6)
TG(O−56:2)
PA(36:4)
PC(33:0)
TG(54:8)
PI(36:2)
TG(47:0)
PC(O−34:0)
TG(57:9)
TG(62:5)
PC(O−38:1)
PE(O−33:2)
SM(d38:0)
TG(53:6)
PE(O−38:2)
TG(60:9)
SM(d45:2)
DG(43:0)
DG(38:6)
TG(48:2)
PC(24:0)
TG(O−56:6)
S1P(18:0)
PC(44:4)
DG(O−40:1)
PE(O−32:2)
SPH(20:1)
PE(O−35:3)
PE(O−41:5)
PE(38:7)
PE(39:6)
DG(41:3)
LPC(24:1)
LPC(16:1)
SM(d42:4)
PC(O−36:0)
PE(O−32:1)
TG(53:2)
TG(62:6)
PC(38:5)
TG(55:5)
PC(31:0)
SM(d45:1)
PC(O−37:6)
PC(42:7)
PC(O−46:6)
SM(t34:1)
SM(d43:2)
TG(O−59:3)
TG(62:13)
PC(38:1)
PE(38:3)
PC(42:6)
PE(32:0)
